# Nonpharmacological intervention therapies for dementia: potential break-even intervention price and savings for selected risk factors in the European healthcare system

**DOI:** 10.1186/s12889-024-18773-7

**Published:** 2024-05-13

**Authors:** Petra Maresova, Lukas Rezny, Petr Bauer, Marian Valko, Kamil Kuca

**Affiliations:** 1 Faculty of Informatics and Management, University of Hradec Kralove, Rokitanského 62, Hradec Kralove, 50003 Czech Republic; 2 Slovak University of Technology, Bratislava, 81237 Slovakia; 3 Biomedical Research Centre, University Hospital Hradec Kralove, Hradec Kralove, 50005 Czech Republic; 4 Andalusian Research Institute in Data Science and Computational Intelligence (DaSCI), University of Granada, Granada, 18071 Spain

**Keywords:** Dementia, Population prediction, Alzheimer’s disease, Parkinson’s disease, Vascular dementia, Amyotrophic lateral sclerosis, Non-pharmacological intervention

## Abstract

**Background:**

New effective treatments for dementia are lacking, and early prevention focusing on risk factors of dementia is important. Non-pharmacological intervention therapies aimed at these factors may provide a valuable tool for reducing the incidence of dementia. This study focused on the development of a mathematical model to predict the number of individuals with neurodegenerative diseases, specifically Alzheimer’s disease, Parkinson’s disease, vascular dementia, and amyotrophic lateral sclerosis. Scenarios for non-pharmacological intervention therapies based on risk factor reduction were also assessed. The estimated total costs and potential cost savings from societal were included.

**Methods:**

Based on demographic and financial data from the EU, a mathematical model was developed to predict the prevalence and resulting care costs of neurodegenerative diseases in the population. Each disease (Alzheimer’s disease, Parkinson’s disease, vascular dementia, and amyotrophic lateral sclerosis) used parameters that included prevalence, incidence, and death risk ratio, and the simulation is related to the age of the cohort and the disease stage.

**Results:**

A replicable simulation for predicting the prevalence and resulting cost of care for neurodegenerative diseases in the population exhibited an increase in treatment costs from 267 billion EUR in 2021 to 528 billion EUR by 2050 in the EU alone. Scenarios related to the reduction of the prevalence of dementia by up to 20% per decade led to total discounted treatment cost savings of up to 558 billion EUR.

**Conclusion:**

The model indicates the magnitude of the financial burden placed on EU healthcare systems due to the growth in the population prevalence of neurodegenerative diseases in the coming decades. Lifestyle interventions based on reducing the most common risk factors could serve as a prevention strategy to reduce the incidence of dementia with substantial cost-savings potential. These findings could support the implementation of public health approaches throughout life to ultimately prevent premature mortality and promote a healthier and more active lifestyle in older individuals.

**Supplementary Information:**

The online version contains supplementary material available at 10.1186/s12889-024-18773-7.

## Background

In the 1990s, numerous studies [1, 2] highlighted the problem of the increasing number of individuals with dementia in developed countries. The increasing efficiency of health care and preventive measures has resulted in increased expenses for health and social systems [3] that are intended to help individuals of all age groups. Studies based on mathematical models or qualified estimates have focused on the prediction of the development of Alzheimer’s disease (AD) within populations [4]. Models of economic burden are also related to the treatment and care of those afflicted by AD. Ikeda et al. [5] examined the economic impacts of the use of donepezil during the mild and moderate stages of AD, while Gustavsson et al. [6] reviewed health economic modelling across the full AD continuum.

Although intensive research is ongoing in the field of AD treatment, U.S. Food and Drug Administration approved Leqembi (lecanemab-irmb) via the Accelerated Approval pathway for the treatment of Alzheimer’s disease, prevention is important. Thus, attention has been focused on the early diagnosis, prevention, and resolution of the onset of AD and, subsequently, on non-pharmacological intervention therapies.

Observational studies have identified a wide range of risk factors for AD. The greatest risk factor for AD is advanced age, as most individuals with AD are aged 65 years or older [7]. Among studies examining the external risk factors for AD, Barnes and Yaffe [8] reviewed evidence from other meta-analytic reviews of seven potential risk factors that include diabetes, hypertension, obesity, physical inactivity, depression, smoking, and low educational attainment. Vellas et al. [9] reported that the European geographical location reflecting differences in culture and in the health care system did not impact AD progression. Norton et al. [10] provided specific estimates of preventive potential by accounting for the association between a set of risk factors, where the relative risk of AD onset was 1.46 for diabetes mellitus, 1.61 for midlife hypertension, 1.60 for midlife obesity, 1.82 physical inactivity, 1.65 for depression, 1.59 for smoking, and 1.59 for low educational attainment. Neil et al. [11], divided nonpharmacological intervention therapies into the three most influential groups with positive impacts on cognitive decline, including physical activities, cognitive training, and a healthy diet. Additionally, recent clinical studies [11] demonstrated the benefits of music therapy for preventing cognitive decline.

Therefore, this study focused on the potential of non-pharmacological intervention therapies and predicted the number of individuals with neurodegenerative diseases, including AD, Parkinson’s disease (PD), vascular dementia (VaD), and amyotrophic lateral sclerosis (ALS), based on reductions in risk factors. Additionally, this study also estimated the predicted cost development and cost savings from societal perspective for the treatment and care of individuals with these conditions.

## Methods

### Study design

This study aimed to estimate the potential cost developments for selected neurodegenerative diseases in the EU and the systemic healthcare burdens that they may represent. We also analysed scenarios based on the results of risk factors that were previously identified in the literature to quantify the long-term potential cost savings. To facilitate this, we developed a mathematical model to predict the presence of neurodegenerative diseases in the population based on demographic data from the EU.

### Model description and data structures

The model comprised of three parts. First, the model inputs were read and assessed for errors and inconsistencies. The primary data structures were created and initialised using available inputs. The middle part was the actual solver that performed the simulation for a given period. Finally, the required outputs were stored based on the user preferences.

Each step of the simulation corresponded to a one-year period. Three types of events occurred during the year. For patients with dementia, the stage can change (transition), new cases of dementia emerge among the previously healthy population (incidence), and some individuals in both groups will die (mortality) according to population projections. The chances of dying for a patient with dementia are higher than those for a healthy person in the same age cohort by a factor termed the (death) risk ratio that is specific for each ty4pe of dementia.

Although individual events occur randomly throughout the year, the model processes them as blocks in the above order (i.e. transition, incidence, mortality) based on statistical likelihoods. Once all three blocks were completed, all individuals advanced by one cohort as the year ended.

To compute the total care cost, we used the total number of individuals with dementia (and its stage, if applicable) and multiplied that value by the corresponding cost category coefficient.

### Input data

AD was characterised by a set of parameters in the model that included a risk ratio of 2 [12, 13] and prevalence ranges starting at 1.1% (the cohort of 65-year olds) to 81% (the cohort 100+) [14]. The incidence rates vary between 0.37% and 16.33% in the same age cohorts, as previously mentioned for prevalence [15]. The model represents three stages of AD (mild, moderate, and severe). To correctly move patients between model stages, we used a transition matrix as described by Davis et al. (Table 4, without inclusion of the mild cognitive impairment pre-stage) [16].

PD was characterised in the model by a risk ratio of 1.41 [17] with a prevalence ranging from 0.113% in the 50-year cohort to 2.953% in the 100+-year cohort [18]. The incidence rate varied from 0.016% with a peak of 0.118% in the 70–79-year cohort to 0.088% in the highest age cohort [19]. The model represented the five stages of the disease according to the Hoehn and Yahr scale. The starting prevalences of the PD stages were set according to Enders et al. at 13% (I), 30% (II), 35% (III), 17% (IV), and 4% (V) [20]. To correctly move patients between model stages, we used a transition matrix accordingly to keep the patients stable in their respective stages.

VaD was characterised in the model by a risk ratio of 2.27 [21] with a prevalence range of 0.275% in the cohort of 65-year-olds to 4.75% in the cohort aged 100 + years [22]. The incidence rate varied between 0.336% and 6.2% in the ≥ 100-year cohort [23]. The model did not represent any stage of VaD due to the unavailability of data.

ALS was characterised by a risk ratio of 1.57 [24] with a prevalence ranging from 0.0052212% in the 40-year-old cohort to 0.0185261% in the highest age cohort of 100 + years. The peak in the 65–69-year-old cohort was at 0.094%. The incidence rate varied between 0.00251975% and 0.02532564% in the 75–79-year-old cohort [24]. The model did not represent any stage of ALS due to the unavailability of data (Table 1).

**Table 1:** Overview of model calibration. AD, Alzheimer’s disease; PD, Parkinson’s disease; VaD, vascular dementia; ALS, amyotrophic lateral sclerosis

	Age cohorts in the model	Simulated disease stages	Prevalence [%]	Incidence [%]	Death risk ratio [vs healthy person]
**AD**	65–100+	Mild–Moderate–Severe	1.1–81	0.37–16.33	2
**PD**	50–100+	Hoehn and Yahr stages, 1–5	0.113–2.953	0.016–0.088	1.41
**VaD**	65–100+	-	0.275–4.75	0.336–6.2	2.27
**ALS**	40–100+	-	0.005–0.019	0.002–0.025	1.57

We assessed the cost-saving potential of risk factor reduction by examining the combined effects of relative reductions of risk factors by 5%, 10%, 15%, and 20% in the population per decade for each of the seven risk factors on projections for AD and VaD cases until 2050. The methodology and the risk factor parameters for AD were obtained from a previous study [10]. For VaD, the data were taken from another study [25] where the authors used the same approach (Table 2).

**Table 2:** Relative risk of dementia onset for Alzheimer’s disease (AD) and vascular dementia (VaD)

Risk factor	Relative risk AD	Relative risk VaD
Diabetes mellitus	1.46	2.28
Midlife hypertension	1.61	1.59
Midlife obesity	1.60	1.33
Physical inactivity	1.82	1.61
Depression	1.65	2.92
Smoking	1.59	1.26
Low educational attainment	1.59	2.75

To calculate the overall dementia treatment costs, the model used cost data aggregated at the disease severity/stage levels without differentiating between direct and indirect costs. We have collected data from studies that were stratified into one in three categories, including direct health care costs, direct non-medical costs, and indirect costs (annex 1), and these were further divided into disease stages if reported.

Data sources were national studies [6, 26–46], and therefore, they were further processed before use in the model. The first modification was to control for the different publication years. This was accomplished by multiplying by the change in the labour cost index for the category Q-Human Health and Social Work Activities in the country where the study was performed. Additionally, the model used the resulting costs calculated as population-weighted averages based on the countries in which the studies originated.

#### Cost data

The focus of this study was on EU countries, so in the following section, we have included all EU countries for which we were able to obtain dementia treatment cost data (per capita gross domestic product (GDP) range in the selected subset of countries ranges from 17,670 € in Greece to 44,950 € in Sweden) [47]. For AD, the costs resulting from the data collected in the Czech Republic, France, Italy, Greece, Austria, United Kingdom, Sweden, and Germany yielded results that included 23,002 [12,140–41,144] €, 30,263 [13,735–48,286] €, and 48,320 [21,024–67,104] €, for the mild, moderate, and severe AD stages, respectively.

For PD, the costs resulting from the data collected in the Czech Republic, Finland, Italy, Austria, Portugal, UK, Sweden, and Germany yielded results that included 6,124 [1,000–9,341] €, 10,417 [2,646–11,560] €, and 21,617 [2,800–29,265] €, for PD stages HY1, HY3, and HY5, respectively.

For ALS, the costs based on data collected in Spain, Greece, the Netherlands, and Germany were 57,357 [11,251–78,256] €. For VaD, the costs resulting from data collected in Spain were 5,541 € (Table 3). Details with references are available in Annex 1 as population-weighted mean.

**Table 3:** Care costs for all modelled dementias. AD, Alzheimer’s disease; PD, Parkinson’s disease; VaD, vascular dementia; ALS, amyotrophic lateral sclerosis

Disease	Stage 1	Stage 2	Stage 3	Stage description
**AD**	23,002 €	30,263 €	48,320 €	Mild, Moderate, Severe
**PD**	6,124 €	10,417 €	21,617 €	HY1, HY3, HY5
**ALS**	57,357 €	-	-	No stage level data available
**VaD**	5,541 €	-	-	No stage level data available

The costs in different countries and time periods were compared using the Labour Cost Index: Q-Labour Cost Index-Human health and social work activities (Nace-Rev.2 section Q) (2016 = 100) and are used to recalculate the default cost settings for all dementias after adjustment of various data for which the national studies were published. The total costs for the EU are further calculated as a weighted average (weight is the size of the population) for data from individual countries. Thus, the unit is a constant Euro in 2021.

### Implementation

The model was implemented in python using the NumPy package for scientific computing. The object implementation consisted of two primary classes that included one for the model itself and the other for population projections. NumPy arrays of corresponding dimensions were used to store the vector/matrix data. All arrays focused on dementia revealed ranges limited to age cohorts starting from the initial age at disease diagnosis. The complete lists of numerical parameters and data are as follows (the array dimensions are listed in square brackets):

Baseline/no migration population projections [year, age cohort].

Healthy population (individuals without diagnosed dementia) [years, age cohort].

Cases of dementia by stage [year, age cohort, stage].

Patient population (individuals diagnosed with dementia) [years, age cohort] - sum of overall stages.

Initial age at disease onset, number of stages, and death risk ratio (scalar parameters).

Initial prevalence [age cohort].

Initial relative shares of patients in each stage [stage, age cohort].

Yearly incidence [age cohort].

Stage transition table [stage, stage].

Cost table [stage, cost type].

The scenarios for risk factor reduction involve additional parameters that include the dementia onset risk ratio, the population attributable risk (PAR), and the intended reduction per decade.

#### Demography

The model used both the baseline and non-migration population projections from Eurostat starting from 2019 according to one-year cohorts without the distinction of sex. The baseline projections were used as the actual population data in our model, and the no-migration projections were used only to compute the survival rates for each age cohort based on the type of dementia.

#### Death rates and cohort scaling

The risk ratio r for a given dementia type indicated that an individual with dementia exhibited an average r-fold higher chance of dying within a certain period compared with that of an individual in the same age cohort without a diagnosis.

In the baseline projections, the population cohort “k + 1” for a given year consisted of the “k-th” cohort survivors from the year before and those who migrated in minus those who migrated out. To compute realistic survival/death rates, no-migration projections were required. However, applying the death rates obtained from no-migration projections to the baseline cohorts reduced the size of those cohorts. They then must be scaled back to their predicted size for the next year.

#### Impact of risk factor reduction on the onset of dementia

We derived the formulas for the modified incidence rates of dementia based on the long-term changes in the prevalence of risk factors known to accelerate the onset of AD and VaD. Each individual risk factor *f* includes parameters for prevalence ${p}_{f}$, risk ratio ${r}_{f}$, and population-attributable risk PAR_*f*_ that is the relative proportion of diagnosed dementia cases attributed to*f*.

We denoted the number of individuals without a diagnosis of dementia within the respective age cohort by $H\left(i,k\right)$ or *H*. We further divided this group based on the presence of *f*:

$$H={H}_{n}+{H}_{f}=\left(1-{p}_{f}\right)H+{p}_{f}H.$$

The number of newly diagnosed cases within each cohort was $d=sH,$where $s=s\left(k\right)$ was the relative incidence in that cohort. The incidence rates for individuals with and without *f* were bound by ${s}_{f}={s}_{n}{r}_{f},$ hence:

$$\begin{gathered} d = {s_n}{H_n} + {s_f}{H_f} = {s_n}\left({1 - {p_f}} \right)H + {s_n}{r_f}{p_f}H = \hfill \\\,\,\,\,\,\,\,\,\,\,\,\,\,\,\,\,\,\,{s_n}\left({1 + {p_f}\left({{r_f} - 1} \right)} \right)H. \hfill \\ \end{gathered}$$

The incidence rates were then:

$${s}_{f}=\frac{s}{1+{p}_{f}\left({r}_{f}-1\right)}, {s}_{n} =\frac{{r}_{f}s}{1+{p}_{f}\left({r}_{f}-1\right)}.$$

The diagnosed cases attributable to *f* were expressed as:

$${d}_{f}=\left({s}_{f}-{s}_{n}\right){H}_{f}={s}_{n}\left({r}_{f}-1\right){H}_{f}={s}_{n}\left({r}_{f}-1\right){p}_{f}H,$$

which yielded Levin’s formula:

$$PA{R}_{f}=\frac{{d}_{f}}{d}=\frac{{p}_{f}\left({r}_{f}-1\right)}{1+{p}_{f}\left({r}_{f}-1\right)}.$$

By inverting the relations, the other two unknowns were expressed as:

$${p}_{f}=\frac{PA{R}_{f}\left({r}_{f}-1\right)}{\left({r}_{f}-1\right)\left(1-PA{R}_{f}\right)}, {r}_{f}=\frac{PA{R}_{f}}{{p}_{f}\left(1-PA{R}_{f}\right)}.$$

Multiple risk factors affect the onset of dementia, and the most common are diabetes, midlife hypertension and obesity, physical inactivity, depression, smoking, and low educational attainment. Based on their mutual dependence, the combined PAR estimate was introduced, and this accounted for the combined effect of individual factors.

$$PA{R}_{c}=1-{\prod }_{ }^{ }\left(1-{w}_{f}PA{R}_{f}\right),$$

where the weights ${w}_{f}$ were computed as one minus commonality (the proportion of variance shared with the other factors). The prevalence ${p}_{c}$ and risk ratio ${r}_{c}$ of the combined factor are bound by the same relations as before. By reducing ${p}_{c}$, the incidence of dementia drops linearly by the following factor:

$$\rho =\frac{1+\omega {p}_{c}\left({r}_{c}-1\right)}{1+{p}_{c}\left({r}_{c}-1\right)},$$

where we introduced a damping parameter $\omega$($\omega$=0.8 represents a 20% reduction in ${p}_{c}$). Different scenarios where ${p}_{c}$ is reduced over time by given explicit functions have been studied in the literature, and a geometric decrease by a fixed percentage per decade is the most common observation.

## Results

**Fig. 1:**
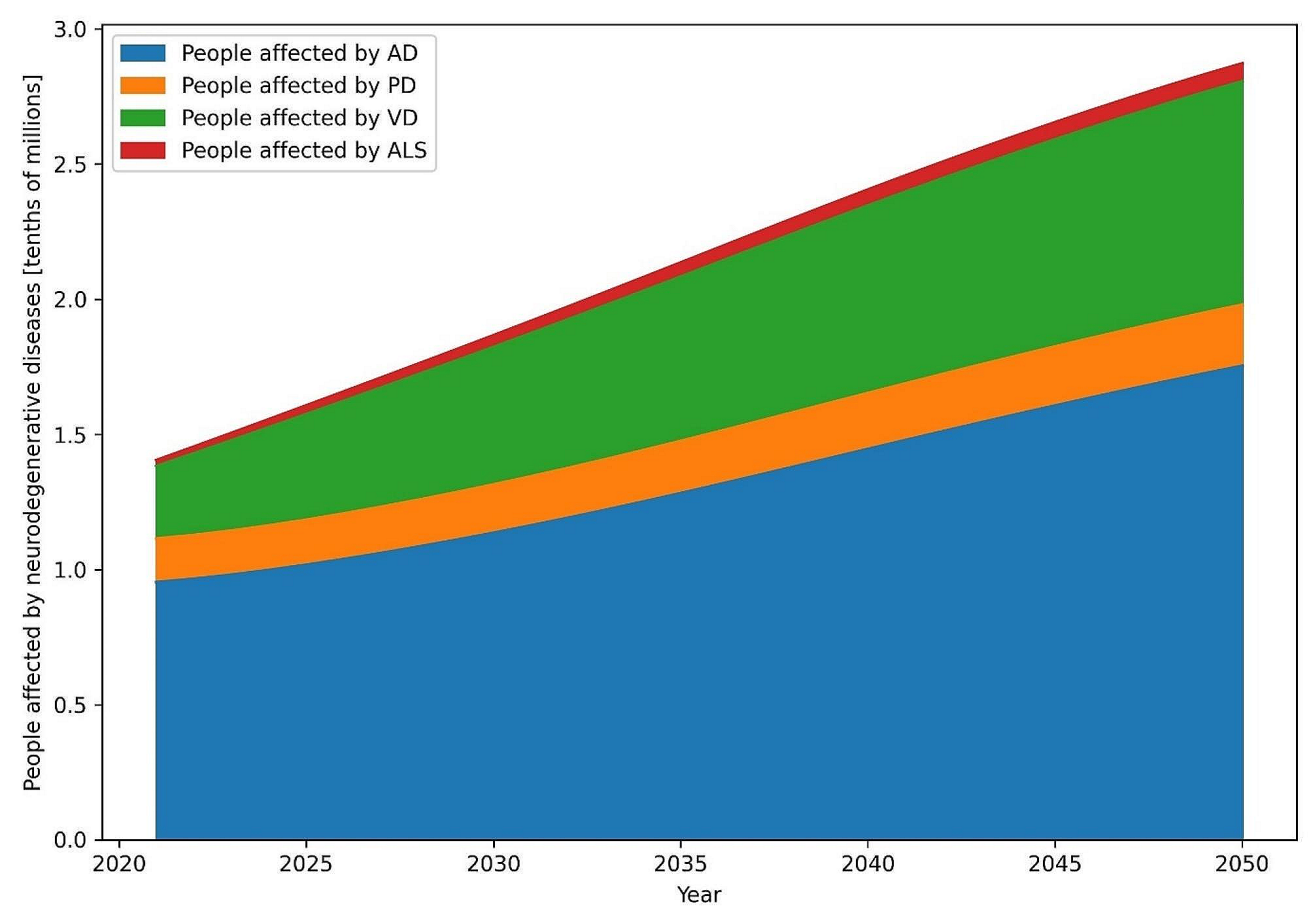
Individuals affected by neurodegenerative diseases in the default scenario without intervention. AD, Alzheimer’s disease; PD, Parkinson’s disease; VaD, vascular dementia; ALS, amyotrophic lateral sclerosis

The model with default parameters presented in the previous chapter revealed that 9.5 million individuals were affected by AD in 2021. This will increase to 17.5 million in 2050, and this represents an 84% growth rate. For PD, an estimated 1.6 million were predicted in the simulation, with an end value of 2.3 million affected individuals in 2050. VD is estimated to exhibit the second-highest number of cases behind AD with 2.7 million affected individuals in 2021 that will increase to 8.3 million cases in 2050. This represents a much faster growth rate compared to that of AD, with the number of cases more than tripling. ALS is overshadowed by previous dementias, as the starting number of cases was estimated to be 0.22 million with an increase to 0.66 million at the end of the simulation (Fig. 1).

**Fig. 2:**
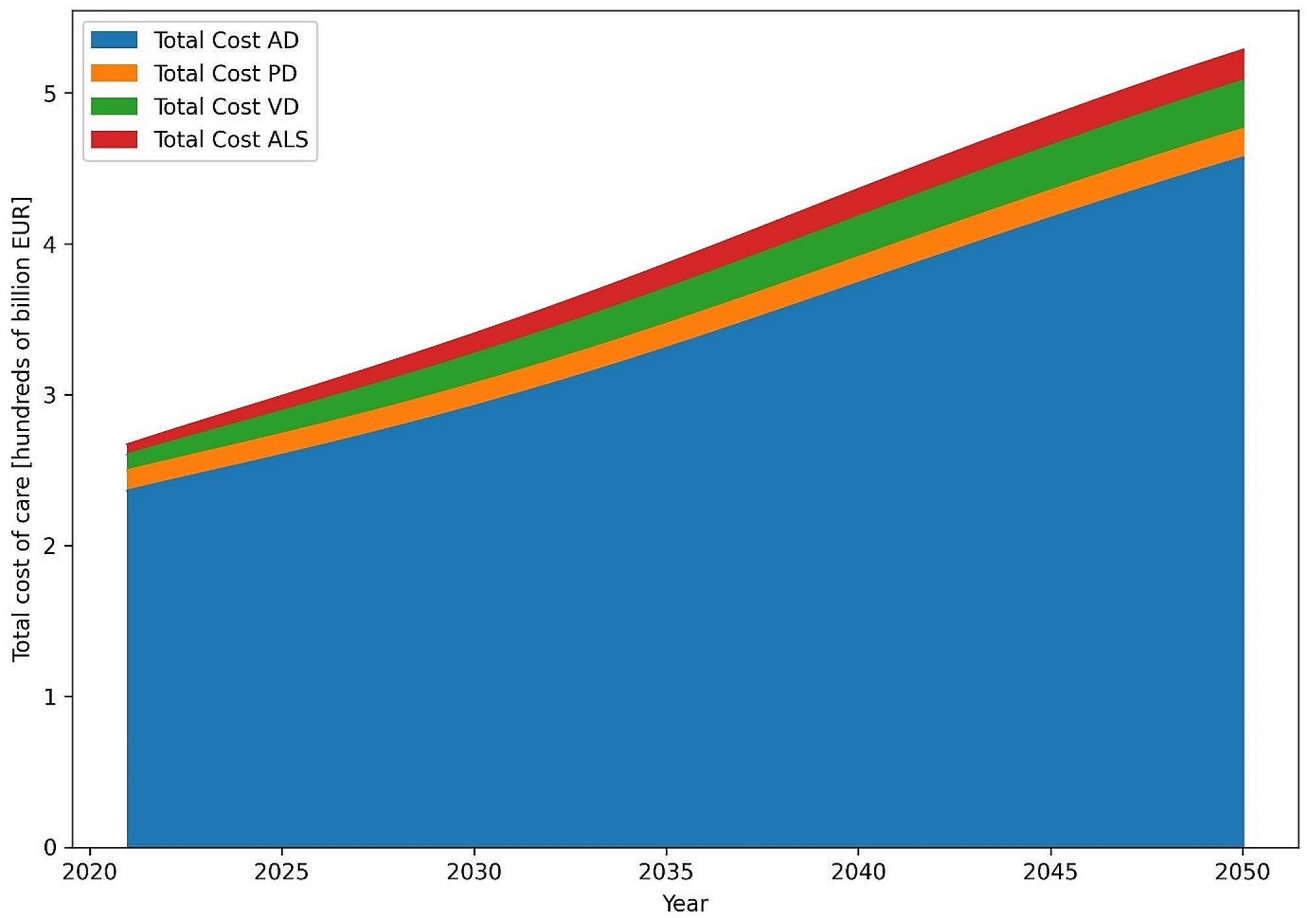
Total costs of care for the treatment of individuals with neurodegenerative diseases. AD, Alzheimer’s disease; PD, Parkinson’s disease; VaD, vascular dementia; ALS, amyotrophic lateral sclerosis

For the data detailing the cost of care for the treatment of selected neurodegenerative diseases, the model demonstrated that there were clearly dominant costs for the treatment of AD with an estimated growth from 236 billion to 457 billion EUR in 2050, and this represented an estimated 88.3% of the total costs in 2021. These decreased to 86.6% in 2050. The PD treatment cost is relatively insignificant with growth from 14 billion EUR in 2021 to 19 billion EUR in the final year of the simulation, and this represented approximately 4% of the treatment cost of total dementias. VD cost start even lower at 10 billion EUR but grow relatively rapidly to 32 billion EUR, thus increasing its total share in treatment cost from 3.8 to 5.9% in 2050. The VD share in treatment cost was in stark contrast to the relatively high total number of individuals burdened by the disease. ALS grew relatively rapidly from 7 billion EUR to 21 billion in 2050, and while its total share in the treatment costs was minor, it was relatively higher than the number of individuals burdened by the disease (Fig. 2).

Table 4 provides a detailed view of the development of costs with classification into direct and indirect costs. The ratio between direct and indirect costs is relatively the same over time. The values ​​for 2050 indicate that for the three groups of costs for all dementias, indirect costs prevail everywhere (more details for all years in Annex 2).

**Table 4:** Predicted direct and indirect costs in 2050. AD, Alzheimer’s disease; PD, Parkinson’s disease; VaD, vascular dementia; ALS, amyotrophic lateral sclerosis

	Direct medical costs	Direct non-medical costs	Direct total costs	Indirect cost
**AD**	478,227,364 €	1,921,008,171 €	8,683,443,278 €	2,399,235,535 €
**VaD**	89,151,695 €	288,068,119 €	587,317,427 €	377,219,814 €
**PD**	61,255,156 €	56,022,179 €	371,229,804 €	117,277,335 €
**ALS**	105,142,171 €	363,859,634 €	220,213,892 €	469,001,805 €

### Intervention scenarios

The model was used to assess hypothetical interventions that could result in a reduction of risk factors influencing the development of AD and VaD. Four scenarios were used in total and simulated the relative reduction of prevalence of the combined risk factor in the population by 5%, 10%, 15%, and 20% per decade, respectively. The estimated savings were discounted by 2% per year to represent the long-term inflation estimate for the Eurozone.

**Fig. 3:**
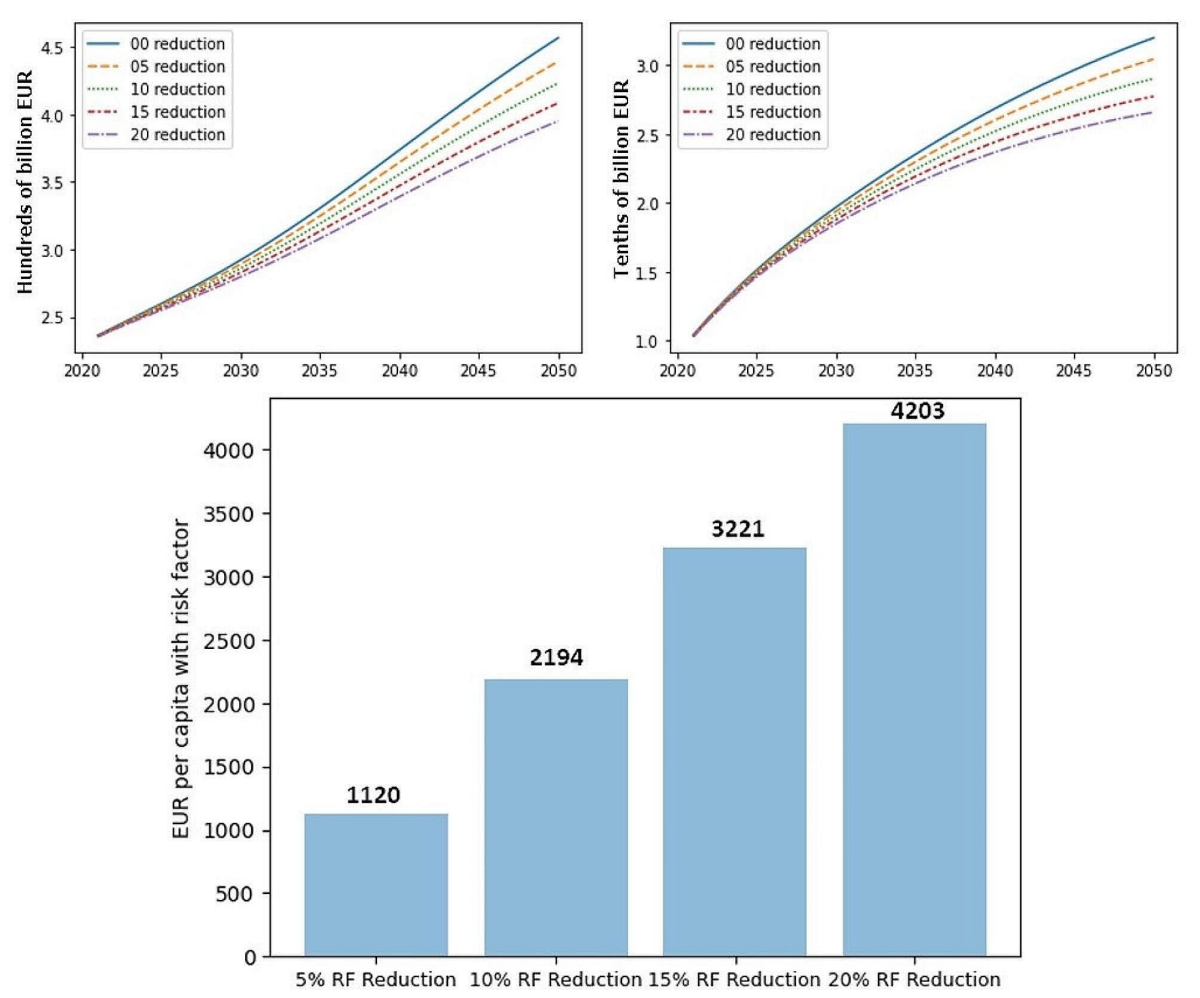
Cost development for selected dementias during the simulation of interventions to reduce risk factors (RFs). Left: Alzheimer’s disease, Right: vascular dementia, Bottom: Break-even intervention cost per capita

The results for AD indicated a maximum discounted savings potential of up to 33 billion EUR (457 vs. 395 billion EUR nominal spending estimate on AD treatment in 2050) in the 20% risk factor reduction scenario and up to 3 billion EUR of discounted savings (32 vs. 27 billion Euro nominal spending estimate on AD treatment in 2050) in the case of VaD and a 20% risk factor reduction. The bottom chart presented in Fig. 3 indicates the estimated break-even intervention costs for all simulated scenarios and represents the maximum break-even intervention cost per capita (calculated as the proportion of the population with risk factors and aged 65 years and older that represented the population eligible for hypothetical intervention).

## Discussion

Our prediction of the number of individuals with dementia corresponded to those of other studies and analyses. Alzheimer Europe (*Prevalence of Dementia in Europe*, n.d.) predicted that the number of individuals with AD will reach 14.298 million in the EU and 18.846 million in the wider European region in 2050. Brookmeyer et al. [48] forecasted a total global burden of AD in 2050 of 16.51 million in Europe, while Nichols et al. predicted 17.819 million patients with AD in Central, Western, and Eastern Europe. Our study predicted 17.5 million individuals with AD in 2050 (an 84% growth rate from 2021), thus revealing a rough concordance with previously published literature based on different methods (see Sect. 2.2).

**Fig. 4:**
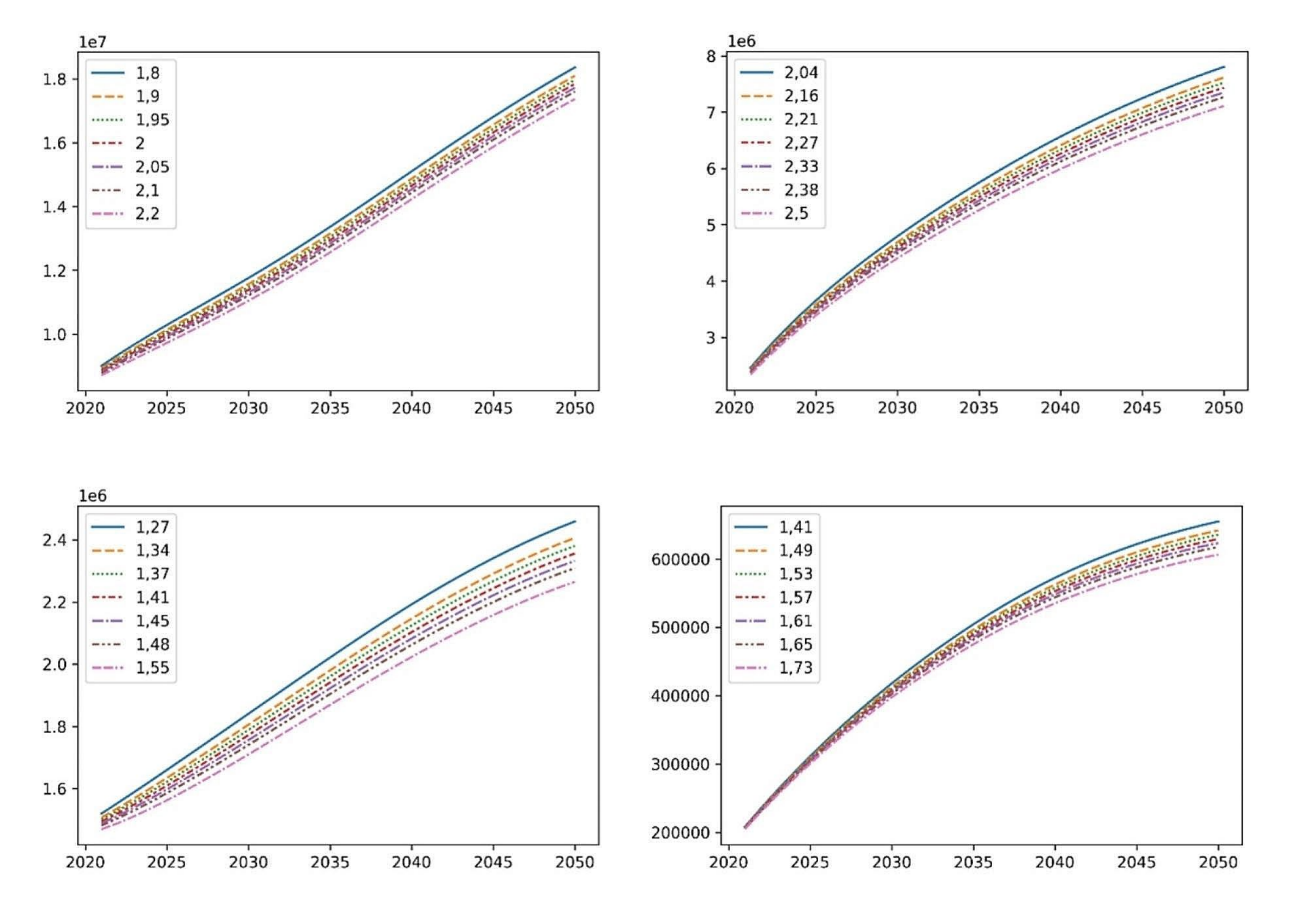
Model sensitivity analysis for death risk ratio (R, values in upper left corner), y axis—total cases of dementia in population; x axis—years; from left to right: Alzheimer’s disease, vascular dementia, Parkinson’s disease, and amyotrophic lateral sclerosis

We performed a sensitivity analysis concerning the key parameter death risk ratio (Fig. 4). For AD, varying this parameter from 1.8 to 2.2 led to relatively small variance of ± 2.8% from the median for the number of cases (18,358,832 to 17,368,855). For VaD, variance was ± 4.7% from the median (7,803,539 to 7,108,134) when the risk ratio varied from 2.04 to 2.5. Observed variance for PD was similar at ± 4.19% from the median (2,458,425 to 2,264,445) for risk ratios of 1.27 to 1.55, and it was ± 3.9% for ALS (654,845 to 606,471) when the risk ratio varied from 1.41 to 1.73. In total, the observed sensitivity of the model is not concerning. Furthermore, the most significant overall result is that the AD affected population exhibits the smallest observed variance (for more details see Annex 3).

Our computations originally assumed that 100% of cases of dementia were identified, thus resulting in a significant overestimation of the direct costs of healthcare that does not occur for undiagnosed cases. The indirect care costs incurred by the families of patients are still valid. Aldus et al. performed a systematic review of the global prevalence of undetected dementia based on 23 studies, and 21 of these were conducted in Europe or North America. The average reported proportion of individuals with undiagnosed dementia was 61.7% [49]. We used this finding to reduce direct medical costs to account for undiagnosed cases of all dementias. Our cost analysis that was performed based on the literature review revealed that the major proportion of care costs for patients with dementia was due to AD, where indirect care costs comprised 73% of the total costs. Thus, the previous overestimation of total costs in our model was approximately 16.7% (Annex 1). After removing non-occurring direct costs for all dementias, the forecasted total costs dropped by 20% overall.

Pedroza et al. [50] also employed various methods to create a model estimating dementia costs globally. The authors used meta-regression to estimate the spending attributable to dementia for those receiving home- and community-based nursing care, and the spending projections from 2020 to 2050 were reported in 2019 United States dollars (PPP). The authors estimated global spending for dementia to be 263 billion USD in 2019, with further growth of up to 1.6 trillion USD in 2050. For comparison to our findings, we converted this value to the unit used in our study (2021 constant Euro). Using a conversion rate of 1.12 EUR/USD (nominal EUR/USD exchange rate as of June 14, 2019, the date related to cost data in the study), we observed global spending and predicted spending of 235 billion EUR and 1.43 trillion EUR, respectively. After adjusting our results by subtracting the indirect costs (73.35% for AD, 63.32% for PD, 25.65% for ALS, and 53% for VaD), the totals were 78 billion EUR for 2021 and 159 billion EUR for 2050. Therefore, our results comprise 33.2% (2021) and 11.1% (2050) shares for the EU in total costs estimated, and this appears to be realistic for an EU population of roughly 447 million individuals that accounts for over 5% of the world population (with limited population growth). Thus, the results obtained based on different methods by other studies were comparable to ours.

We modelled future expected care costs allocated to dementia patients treated at a 2% discount rate to arrive at the current discounted value of the intended intervention cost break-even point. This is precisely the same as the European Central Bank inflation target (2%). This may seem low given the last inflation estimate for the Euro area in March 2022 of 7.5% [51]. However, this variable must be assessed over longer periods, as the current economic climate is highly turbulent and unpredictable. Since 2013, the Eurozone experienced the opposite problem, with the consumer price index repeatedly on the verge of deflation. Therefore, our choice of a 2% rate appears reasonable and is a parameter for which it is not overly complicated to perform a sensitivity analysis of the break-even point of the intervention cost.

### Study limitations

One limitation of the model is the observation that it does not account for the simultaneous occurrence of multiple dementia types for one patient. Literature reports that a typical combination is AD and VaD, with reports of up to three dementias per patient. This condition is referred to as mixed dementia (MD). Custodio et al. reported a 22% share of MD (AD with VaD) among patients with dementia [52]. As the model in the present study accounts for each dementia separately, it overestimates the total number of individuals with dementia in the population as well as the total costs based on the observation that the MD treatment costs are not a simple sum of the respective partial costs. Currently, when information regarding updated medication recommendations and their costs is lacking, it is not possible to estimate the degree of overestimation by our model.

In this study, cost predictions for ALS in particular and somewhat also for VAD must be considered as highly uncertain. While we identified 9 national expenditure studies for AD and 8 for PD, the number of studies declined to 4 for VAD and only one from Spain regarding ALS care costs.

Our study assumes a causal relation between risk factors and dementia onset based on observational cohort studies. It is important to note that there is at best mixed evidence for this based on randomized clinical trials which we discuss further in the following section.

The Finnish Geriatric Intervention Study to Prevent Cognitive Impairment and Disability (FINGER) was designed to assess a multidomain approach to prevent cognitive decline in at-risk older individuals from the general population. The study enrolled 1,260 individuals aged 60–77 years (mean age of the participants was 69.3 years), and inclusion criteria included the presence of cardiovascular disease, advanced aging, dementia risk factors, and cognition at mean level or slightly lower than expected for their age. The intervention group received nutritional intervention and a physical exercise training programme incorporating strength and aerobic training, and cognitive training and social activities were encouraged through group meetings in previously mentioned intervention activities. The primary results of the trial demonstrated that after two years, the cognitive performance in the intervention group was better, and their risk for cognitive decline was lower [53]. Unfortunately, the study interpretation was difficult, as the control group that received standard care also exhibited cognitive improvements that may be the result of repeated exposure to the standardised cognitive testing. The authors planned to perform a seven-year follow-up that unfortunately has not been published yet [54].

The Dutch Prevention of Dementia by Intensive Vascular Care assessed if a multidomain intervention targeting of cardiovascular risk factors can prevent dementia in a population of older individuals (individuals aged 70–78 years were recruited through general practices in the Netherlands with a total of 1,890 participants in the intervention group). Participants were assigned to either a 6-year nurse-led, multidomain cardiovascular intervention or the control group where they received standard care. The intervention consisted of visits to a practice nurse for a period of six years with 18 visits in total. During these visits, the nurse assessed cardiovascular risk factors that included smoking, diet, physical activity, weight, and blood pressure. Blood glucose and lipid concentrations were assessed every two years. Based on these assessments, individually tailored lifestyle advice was provided. Long-term nurse-led vascular care in an unselected population of community-dwelling older individuals did not result in a reduction in the incidence of all-cause dementia, disability, or mortality. The authors concludes that in health-care systems with high standards of typical care, such as those in the Netherlands, the potential for preventing dementia by improving cardiovascular risk factor management may have been too small to observe significant beneficial effects [55].

The MAPT study (Multidomain Alzheimer Preventive Trial) was a three-year, randomized, placebo-controlled trial that was followed by a two-year observational extension. All participants were individuals without dementia that were randomly assigned (1:1:1:1) to the multidomain intervention plus omega 3, multidomain intervention plus placebo, omega 3 alone, or placebo alone groups. The objectives were to assess the cognitive effect of MAPT interventions in individuals with non-dementia. The multidomain intervention (with 837 total participants in both multidomain groups) included training sessions in three areas that included nutrition, physical activity, and cognitive training. Training sessions were conducted in 120 min sessions with each session including 60 min of cognitive training, 45 min of physical training, and 15 min of nutritional advice. Training frequency was two sessions each week for the first month and one session each week for the second month. From the third month, the frequency declined only to one 60-minute session each month throughout the three-year intervention period [56]. The multidomain intervention and polyunsaturated fatty acids (either alone or in combination) exerted no significant effects on cognitive decline over three years in older individuals with memory complaints [57]. This negative result may be due the lower frequency of the non-supervised physical exercise when compared to that of the previously cited FINGER study.

To sum up the studies, the Dutch Prevention of Dementia by Intensive Vascular Care did not include directed active intervention in the form of physical exercise, cognitive training, or social activities. The MAPT study included two sessions initially, but for the vast majority of the study there was only one monthly combined training session. The results of both studies were negative. In contrast, the FINGER study contained supervised physical exercise at least thrice a week that included both strength and aerobic activities [53]. It was concluded that the FINGER study supports the efficacy of multidomain prevention approaches, as the improvement was significantly greater in the intervention group compared with that in the control group. A common shortcoming of the aforementioned studies is that the intervention occurs in an already older population (mean enrolment age of approximately 70 years for all studies). Studies including younger populations would necessitate longer follow-ups, and the issue then arises of the feasibility of conducting such large and long-term trials.

It could be argued that the evidence is too weak to support the recommendation of lifestyle interventions targeted to dementia specific risk-factors and that on top of that, that those lifestyle interventions incur additional costs which are unaccounted for in our model. According to Food and Agriculture Organization (2022) [58] of the United Nations, average cost of a healthy diet in 2020 was 3.54 USD per person and day, which is an price unaffordable by 42% of world population. AD is currently less of a concern in developing countries with significantly lower life expectancies compared to developed ones. For developed countries, 3.54 USD cost of healthy diet per day is significantly less than the price of popular product of McDonalds, its iconic Big Mac, which costs 5.69 USD in USA and 5.39 EUR in EU respectively and which represents only single meal on top of that. Therefore, it could be argued that healthy diet can be even cheaper compared to diet composed processed fast-food meals for population of rich, developed countries. Regarding lifestyle interventions focused on physical fitness, raw strength and cardiovascular conditioning [59], here again, costs can be very low. Cardiovascular condition can be improved and maintained with jogging, running or biking perfectly well. Strength training can be performed with one’s own bodyweight necessitating nothing more than a bar, perhaps supplemented with cheap pair of gymnastics rings and Kettlebell, tools known to man for more than a century. Aforementioned activities can be done both solitarily and, in the sports clubs, which provides further avenues for socializing. Biking or walking to work if possible, is once again cost saving compared to habit of commuting to work with one’s own car, as is often done in developed rich countries. In overall, lifestyle interventions focused on dementia risk factors can be implemented in a cost-saving way.

### Future directions

The potential for more accurate modelling lies in the availability of data. Several of the advanced features are even implemented but were not used in the actual simulations. For example, the death risk ratio does not depend on the stage of dementia (for AD, PD), and this likely leads to overestimation of the share of patients in more severe stages.

On top of using a single value of death risk ratio (*R* = 2.0 for AD) regardless of stage, we tried using values of R based on [13]. Unfortnately, the study does not contain relative risks based on the stage of AD, but rather just based on its duration in years prior to death. These of course do not correspond on one-on-one basis, but are the best we could find. Same as in other simulations, we took the initial relative shares of AD patients 67%, 22% and 11% for mild, medium and severe stage respectively. The corresponding relative death risks of R1 = 1.73, R2 = 2.43, R3 = 2.79 were scaled to give the weighted average R_avg∼2.0 (the study itself states that the overall unadjusted relative risk associated with a diagnosis of AD is 2.03). Throughout the entire simulation, we observed higher share of patients in severe stage compared to applying R_avg regardless of stage; see the results in the Annex 4.

We also tested a hypothetic scenario that would keep the relative shares of patients constant through the simulation. This required the ratios of 1.56 : 1.76 : 4.48 (before scaling). With lower relative value of R3, the share of patients in severe stage was increasing. The stage-based risk ratios proved difficult to obtain on national scale which is what we would ideally want. The age-specific values for relative risks are also provided in [13], but only for three age groups (age < 80, 80–89, age > 90), rather than 1-year cohorts we use in our model.

The model could also include the phase that precedes the onset of dementia that is commonly referred to as mild cognitive impairment. Another direction could be modelling simultaneous occurrence of multiple dementia types or scenarios linked to current solutions in diagnosis or treatment of diseases.

Regarding the intervention scenarios, most of the risk factors for dementia onset also heavily influence the overall death rates. Reducing their prevalence in any significant manner, such as promoting a generally “healthier” lifestyle, would mean more survivors in the later age cohorts and possibly higher absolute numbers of dementia cases. This was already mentioned in a previous study [10] and would require a complex system dynamics model implementing this feedback loop and all related logic, whereas at the current state of the model, the population data are exogeneous quantities. Such a task is beyond the scope of this study and may be the subject for future research.

## Conclusion

Our model is unique in that it represents a bottom-up approach for modelling future population dementia dynamics. The model was validated by comparisons to previously cited statistical estimates of future population prevalence [25] and meta-regression cost estimates [27], where we obtained comparable results to those in the cited studies. The added value of the created model lies in the possibility of detailed testing of “what if” scenarios, as it uses detailed parametrization of selected dementias and can therefore be used to model impacts and costs of various intervention scenarios, thus providing early feedback and cost-benefit analysis as was presented in this study examining the general interventions aimed to reduce risk factors. Overall, we constructed a replicable mathematical model to predict the prevalence and resulting cost of care of neurodegenerative diseases in the population. The potential cost savings and predicted number of individuals with neurodegenerative diseases due to risk factor reduction may inform healthcare policy decisions regarding the allocation of resources for neurodegenerative disease prevention and treatment.

While being relatively complex, the model uses various simplifications that are usually due to the lack of reliable data to back up more exact modelling. Healthcare data possesses the potential to transform our understanding of health, disease, and outcomes, yet it is currently scattered across multiple institutions and countries, stored in different formats, and subject to different rules. If more data is available for future research, the model could be developed to consider more specific variables, and it will bring more accurate results and will be able to be used more for qualified decisions.

### Electronic supplementary material

Below is the link to the electronic supplementary material.

Supplementary Material 1

Supplementary Material 2

Supplementary Material 3

Supplementary Material 4

## Data Availability

The datasets used and/or analysed during the current study are available from the corresponding author on reasonable request. A portion of the data is included in the Annex.
